# Cardiopulmonary alterations by ultrasound in a patient with uncomplicated mixed malaria infection: a case report from the Amazon Basin

**DOI:** 10.1186/s12936-021-03861-5

**Published:** 2021-07-28

**Authors:** Alma Wegener, Karine O. Lima, Anna E. Holm, Laura C. Gomes, Luan O. Matos, Isabelle V. M. Vieira, Rodrigo Medeiros Souza, Claudio Romero Farias Marinho, Lasse S. Vestergaard, Tor Biering-Sørensen, Odilson M. Silvestre, Philip Brainin

**Affiliations:** 1 Multidisciplinary Centre, Federal University of Acre, Câmpus Floresta, Cruzeiro do Sul, Acre, Brazil; 2 Department of Cardiology, Herlev and Gentofte Hospital, Cardiovascular Non-Invasive Imaging Research Laboratory, University of Copenhagen, Hospitalsvej 8, Post 835, 2900 Copenhagen, Denmark; 3 Department of Parasitology, Institute of Biomedical Sciences, University of São Paulo, São Paulo, Brazil; 4 National Malaria Reference Laboratory, Department of Bacteria, Parasites and Fungi, Statens Serum Institut, Copenhagen, Denmark; 5 Faculty of Biomedical Sciences, Copenhagen University, Copenhagen, Denmark; 6 Health and Sport Science Center, Federal University of Acre, Rio Branco, Acre, Brazil

**Keywords:** Malaria, Heart disease, Pericardial effusion, Pericarditis, Echocardiography, Lung ultrasound

## Abstract

**Background:**

Information on cardiopulmonary complications in clinical malaria is sparse and diagnosis may be difficult in resource-limited areas due to lack of proper diagnostic tools and access to medical care. A case of pericardial effusion and pulmonary alterations assessed by ultrasound in a patient with uncomplicated mixed malaria infection is described.

**Case presentation:**

A previously healthy 23-year-old male from the Amazon Basin was diagnosed with mixed infection of *Plasmodium vivax* and *Plasmodium falciparum* by peripheral blood smear. The patient presented with mild malaria symptoms without signs of severe malaria, but reported moderate chest pain and shortness of breath. Laboratory analyses revealed thrombocytopenia and anemia. The electrocardiogram had PR depressions and bedside ultrasound of the cardiopulmonary system showed pericardial effusion (18 mm) accompanied by multiple B-lines in the lungs, identified as vertical artifacts extending from the pleural line. Cardiac biomarkers were normal. The patient was treated according to national guidelines for malaria and suspected pericarditis, respectively. At follow-up on day 5, the pericardial effusion (9mm) and B-lines had markedly decreased. By day 21 the patient was asymptomatic, had completed the treatment, and the electrocardiogram and ultrasound findings had normalized.

**Conclusions:**

This case report highlight the usefulness of bedside ultrasound to identify cardiopulmonary involvement in patients with uncomplicated malaria and relevant symptoms.

## Background

Information on cardiopulmonary complications in malaria is limited and only sporadically reported in clinical studies [1, 2]. Adequate and timely diagnosis of these complications may be difficult in low- and middle-income countries where access to healthcare facilities and diagnostic imaging tools are limited. Bedside ultrasound examination, including echocardiography and lung ultrasound (LUS), is a non-invasive tool that can detect cardiopulmonary alterations [1]. Both modalities may be conducted outside of clinical wards and has previously been demonstrated useful in resource limited settings [2–4]. Pericardial effusion by echocardiography has been described in several reports of *Plasmodium vivax* and *Plasmodium falciparum* [5–11], but is not routinely assessed in patients with malaria. B-lines assessed by LUS are vertical artifacts starting from pleura and are caused by an increase in interstitial density. They are associated with cardiac and pulmonary conditions [1, 12] and one prior study has reported on B-lines in patients with malaria [2].

A case of pericardial effusion and B-lines in a patient with uncomplicated mixed malaria infection, who presented with chest pain and shortness of breath is reported. The aim of this case report is to describe the potential usefulness of ultrasound for identifying cardiopulmonary complications in relevant patients suffering from uncomplicated malaria.

## Case presentation

A 23-year-old Brazilian male with no prior history of cardiopulmonary disease was diagnosed with *P. vivax* and *P. falciparum* by peripheral blood smear (16560 parasites/µL) in the emergency care unit (UPA, Cruzeiro do Sul, Acre, Brazil). 48 days earlier he had completed treatment for mono-infection of *P. vivax* (peripheral blood smear, 300 parasites/µL) with primaquine 30 mg (7 days) and chloroquine 600 mg (day 1) + 450 mg (day 2 and 3). The patient now presented with headache and chills and reported onset of stabbing chest pain and shortness of breath 3 days prior to examination.

**Fig. 1:**
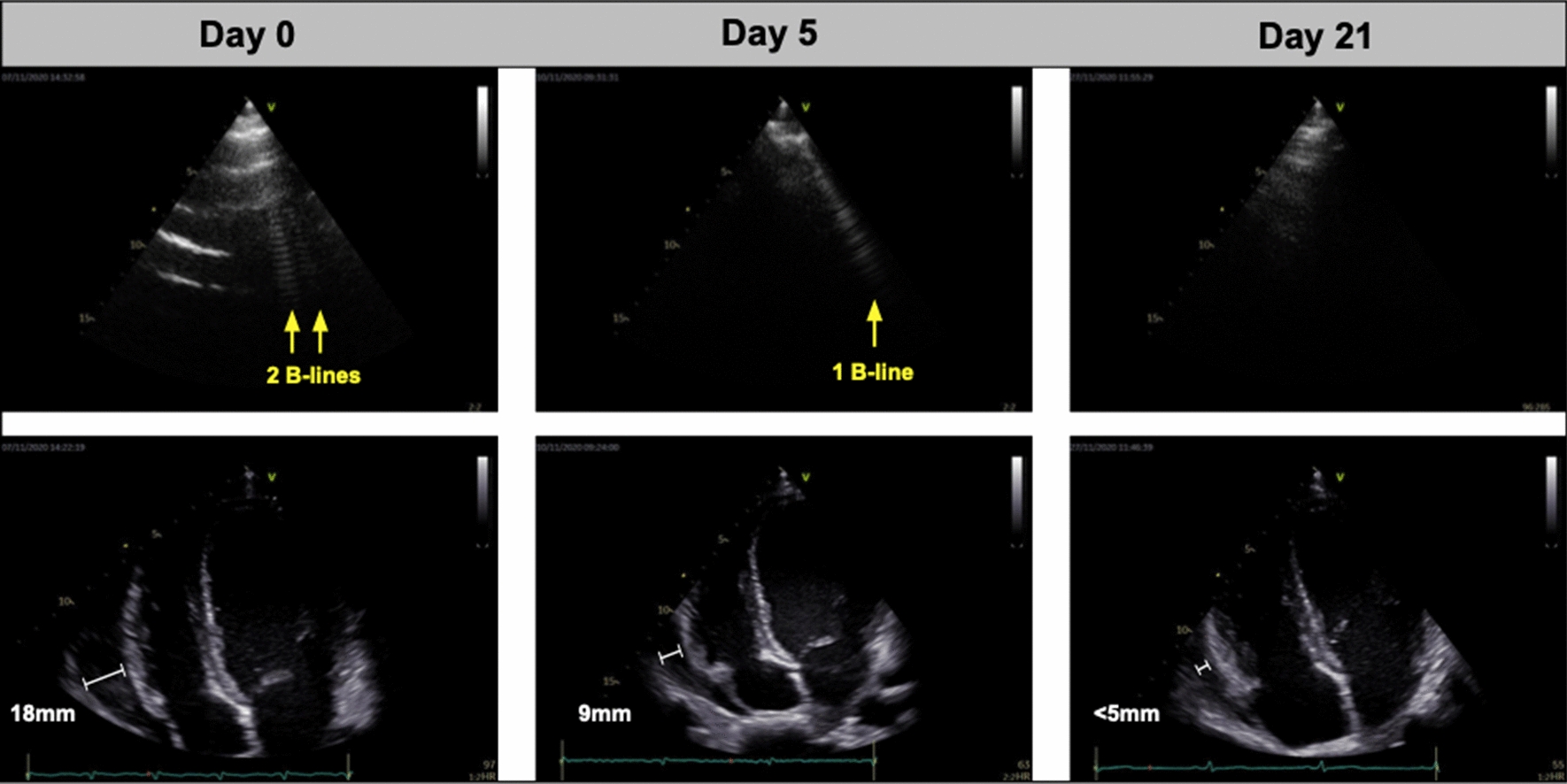
Images of cardiopulmonary complications in malaria by day 0, 5 and 21. Upper row shows B-lines by lung ultrasound (white artefacts extending from the pleura) and the lower row displays pericardial effusion measured next to the right ventricle

 The patient was circulatory stable (blood pressure 110/62 mmHg; heart rate 109 beats/min), auscultation of heart and lungs was normal. Axillary temperature was < 37.5 °C. Laboratory results showed inflammation (C-reactive protein 96 mg/L), thrombocytopenia (platelets 99/mm^3^) and moderate anemia (hemoglobin 9.2 g/dL). Liver function was slightly affected (aspartate aminotransferase 47 U/L). Cardiac biomarkers were all within normal range (pro-brain natriuretic peptide 24 pg/mL; troponins < 0.001 ng/mL). The electrocardiogram (ECG) showed PR-depressions in lead I, II, aVF and V1–V6. Ultrasound revealed pericardial effusion (18 mm) and 24 B-lines by LUS (Table 1; Fig. 1), which was assessed as the sum of B-lines in eight thoracic zones. All other echocardiographic measurements were within normal range, including left ventricular ejection fraction (> 50 %) and right ventricular function and no pleural effusions were found. Test for coronavirus by polymerase chain reaction was negative. The patient started treatment the same day with (i) primaquine 30 mg (14 days) (ii) artemether 80 mg + lumefantrine 480 mg × 2 (3 days) and (iii) colchicine 0.5 mg × 2 + ibuprofen 600 mg (7 days).

**Table 1:** Overview of ultrasound findings and biochemistry at day 0, 5 and 21

	Day 0	Day 5	Day 21
Lung ultrasound			
B-lines (no.)	24	6	0
Cardiac ultrasound			
Pericardial effusion (mm)	18	9	< 5
Left ventricular ejection fraction^a^ (%)	53	53	53
Fractional area change (%)	55	50	50
Pulmonary artery systolic pressure (mmHg)	17	17	20
Biochemical findings			
Hemoglobin (g/dL)	9.2	9.2	12.7
Platelets (/mm^3^)	99	144	248
C-reactive protein (mg/L)	96	33	< 10

^a^Estimated by Simpson’s biplane method

Follow-up day 5: Chest pain and shortness of breath improved after initiation of treatment and the patient reported to be compliant with the medication. The ECG showed sinus rhythm without PR-depressions. The pericardial effusion was reduced to 9 mm and 6 B-lines were observed. Blood sample analyses showed a decrease in C-reactive protein (32 mg/L) and peripheral blood smears were negative for *Plasmodium* species.

Follow-up day 21: The patient reported to be free from all symptoms and peripheral blood smears were negative. Ultrasound displayed no significant pericardial effusion (< 5 mm) and 0 B-lines.

## Discussion

 This case report has two principal findings: (i) Malaria patients who present with cardiac complaints may suffer from unrecognized cardiopulmonary complications, and (ii) bedside ultrasound examination may be a useful method to identify such cardiac disturbances. Recently, a meta-analysis by Holm et al. found that cardiovascular complications in malaria are only sporadically reported and may be underestimated [13]. Specifically, ECG and left ventricular dysfunction were among the most prevalent complications associated with malaria. As well, a clinical study from the Amazon Basin found that non-severe *P. vivax* infection (n = 26 outpatients) was associated with subclinical right ventricular function (i.e., lower fractional area change) in the symptomatic phase of the malaria infection [14]. In this case, left and right ventricular function were normal at baseline and at follow-up (Table 1). By contrast, PR-depressions were seen in the ECG and pericardial effusion in the absence of myocardial affection, thus implying pericarditis [15, 16]. Colomba et al. reported similar findings in a malaria case and suggested that local inflammation might increase vascular permeability, leading to accumulation of pericardial fluid [6]. Other suggested mechanisms include cytoadhesion of infected erythrocytes to the coronary endothelium [17] and release of pro-inflammatory cytokines due to vascular endothelial dysfunction in the myocardial vessels [18, 19]. Despite a plethora of proposed mechanisms, no consensus exists as to how malaria affects the heart.

Inflammatory changes also occur in the lungs during malaria [20, 21]. Although pulmonary edema represents a criteria for severe malaria infection, and is associated with high mortality, lung complications are also seen in uncomplicated cases [20, 22]. Lung impairment may appear in mono-infections of *P. vivax* and *P. falciparum*, however, occurs more frequently in mixed malaria infections [23]. In the present case it is possible that the patient had a relapse of *P. vivax* followed by a superinfection of *P. falciparum*, increasing the risk of pulmonary involvement. Consequently, multiple B-lines by LUS were observed, indicating presence of extravascular lung water [23]. One prior clinical study also reported B-lines in malaria patients, however, the sample size was small (n = 31), the population consisted of adults and children, and no relationship was found with degree of malaria severity, which was defined by a combination of clinical parameters and high parasite density (parasite count > 100,000/mm) [2].

During the follow-up examinations, the patient reported a relief in cardiac complaints which was accompanied by a concomitant decrease in pericardial effusion and B-lines. This indicates a correlation between cardiac symptoms and cardiopulmonary findings by ultrasound. Malaria patients presenting with key cardiac symptoms (i.e., chest pain, shortness of breath) could potentially constitute a *high-risk* group, who may benefit from ultrasound of the cardiopulmonary system as part of the early clinical assessment. Furthermore, B-lines demonstrated a dynamic nature throughout the follow-up examinations and could, on a hypothesis-generating basis, be used for monitoring of pulmonary involvement in malaria; similar to what has been reported in other patient groups such as heart failure and sepsis [2, 12, 23].

Bedside ultrasound examination represents an easily applicable diagnostic tool, which may provide accurate information for diagnosis of cardiopulmonary involvement in clinical malaria cases. LUS may be particularly useful as it can be applied by non-medical personnel with high sensitivity and specificity [24], and even is applicable in resource-limited settings [4]. Timely use of ultrasound in relevant patients with malaria may possibly facilitate risk stratification, therapeutic decisions and effective management and should be explored in future prospective cohort studies of malaria patients.

## Conclusions

This case report highlights the usefulness of bedside ultrasound to identify cardiopulmonary involvement in patients with uncomplicated malaria and relevant symptoms.

## Data Availability

The datasets used and analyzed during the current study are available from the corresponding author in reasonable request.
